# Capsaicin inhibitory effects on *Vibrio cholerae* toxin genes expression 

**Published:** 2019

**Authors:** Soroor Erfanimanesh, Gita Eslami, Arezou Taherpour, Ali Hashemi

**Affiliations:** 1 *Infectious Diseases and Tropical Medicine Research Center, Shahid Beheshti University of Medical Sciences, Tehran, Iran*; 2 *Department of Microbiology, Kurdistan University of Medical Sciences, Sanandaj, Iran*; 3 *Department of Microbiology, School of Medicine, Shahid Beheshti University of Medical Sciences, Tehran, Iran*

**Keywords:** Vibrio cholera, Antivirulence agents, Capsaicin, Toxin gene expression

## Abstract

**Objective:**

Cholera is an acute secretory diarrhea caused by the Gram-negative bacterium, *Vibrio cholerae *mostly through production of cholera toxin (CT) and zonula occludens toxin (Zot)*.* Isolates of *V. cholerae* have acquired resistance elements during the last decade. One of the most promising ways to treat resistant strains is to use antivirulence agents instead of killing the causative agent with conventional antibiotics. In this study, we examined whether different concentrations of capsaicin - the pungent fraction of red chili- can act as an antivirulence agent and inhibit *V. cholerae* toxin production.

**Materials and Methods:**

Two standard strains namely, *V. cholerae *ATCC 14035 and *V. cholerae* PTCC 1611 were used in this study. Minimum Inhibitory Concentration (MIC) of capsaicin was determined by broth microdilution method. Based on MIC results, the bacteria were cultured in the presence of sub-MIC concentrations of capsaicin and a negative control without capsaicin. Real-time PCR (RT-PCR) was carried out to determine the expression level of *V. cholerae *toxin genes at each concentration.

**Results:**

MIC test showed that 200 mg/mL of capsaicin in 2% dimethyl sulfoxide (DMSO) could inhibit the growth of the two standard strains of *V. cholera*e. The expression of *V. cholerae *toxin genes was significantly reduced following treatment with sub-MIC concentrations of capsaicin as assessed by RT-PCR.

**Conclusion:**

Capsaicin showed great inhibitory effect against cholera toxin and reduced Zot production in the tested strains of *V. cholerae*. The results showed promising insights into antivirulence effects of capsaicin.

## Introduction

Cholera is an acute secretory diarrhea caused by the Gram-negative bacterium *V. cholerae *(Laxminarayan et al., 2013 ▶). WHO reported an increasing trend in cholera outbreaks in the past few years. Approximately 600,000 cases of cholera and 8,000 deaths were reported to WHO in 2011, which are nearly double the frequency reported in 2010 (Sack et al., 2004 ▶). *V. cholerae *is classified into more than 200 serogroups based on the O antigen of the lipopolysaccharide; among these types, only O1 and O139 serogroups cause epidemic form of cholera. *V. cholerae *O1 is classified into two biotypes namely, classical and El Tor (Allen et al., 2014 ▶; Harris et al., 2012 ▶). Ogawa, Inaba and Hikojima are three major serotypes of each serogroup, which vary in prevalence with time. Pathogenesis of cholera involves synchronized expressions of several virulence factors by *V. cholerae*. The two most important virulence factors are toxin-coregulated pilus (TCP) and cholera toxin (CT) (Muanprasat and Chatsudthipong, 2013 ▶) encoded by *tcp*A and *ctx*AB genes, respectively (Chatterjee et al., 2010 ▶). CT is an AB5 toxin type that accounts for severe diarrhea symptoms of cholera. The pentamer form of B subunit binds to the ganglioside GM1 on eukaryotic cells and intracellular translocation of the A subunit occurs; then, it acts enzymatically to activate adenylate cyclase and raise intracellular cyclic AMP. The consequences would be chloride secretion through the apical chloride channel and secretory diarrhea (Harris et al., 2012 ▶). One of the other toxins secreted by *V. cholerae* is Zot, which disturbs small-intestinal tight junction permeability through a protein kinase C–mediated actin polymerization (Fasano et al., 1995 ▶). Similar to other bacteria, some isolates of *V. cholerae* have acquired resistance elements that were found in almost all of the isolated strains during the past decade (Kitaoka et al., 2011 ▶). Antibiotic resistance among *V. cholerae *species was rare before 1977, but after that conjugative plasmid-mediated multiply antibiotic-resistant *V. cholerae *O1 (MARV) emerged as a major problem in Tanzania and then in Bangladesh. Reports from several cholera endemic countries of strains resistant to the commonly used antibiotics including ampicillin, tetracycline, streptomycin, kanamycin and gentamicin have emerged during the past two decades (Sack et al., 2004 ▶). Although it is clear that antibiotics aimed at cellular viability have been historically highly effective, these modes of action enforce selective pressure that adopts the growth of antibiotic-resistant strains (Harris et al. 2012 ▶). Over the past 40 years, with the exception of daptomycin and linezolid, no new classes of antibiotics have been discovered (Silver, 2011 ▶). The increasing trend of antibiotic resistance on one side and our frailty to develop new efficient antibiotics on the other hand, had led to search for novel drug development approaches in order to combat invading bacteria. One of the most promising ways that is increasingly being explored is targeting bacterial virulence factors or disrupting the interaction between the host and the pathogen instead of killing the infection causing bacteria (Roca et al., 2015 ▶). Lots of compounds have been screened for their probable capacity to inhibit pathogens’ virulence factors. S-CMC for example was found to inhibit pneumococcal adherence to host cells (Sumitomo et al., 2012 ▶). Screening of a 50,000-compounds library was used to find a small molecule 4-[N-(1,8-naphthalimide)]-n butyric acid, named virstatin, that could prevent the expression of the two fundamental *V. cholerae* virulence factors, cholera toxin and the toxin-coregulated pilus (Rasko and Sperandio, 2010 ▶). In addition to synthetic chemical molecules, natural compound like ginger (*Zingiber officinale*) and capsaicin, the active component of chili peppers, belonging to the genus* Capsicum* have also shown promising antivirulence activity (Ahmad et al., 2015 ▶; Jensen et al., 2003 ▶). Capsaicin (8-methyl-N-vanillyl-6-nonenamide) is the pungent fragment of *Capsicum* plants (chili peppers), which has been long used as a spice in many countries, including Iran (Zhang et al., 2017 ▶). Capsaicin and related compounds –capsaicinoids- are secondary metabolites of chili peppers which play an important role in plant defense, probably as repellents against animals (Marini et al., 2015 ▶). Furthermore, besides its multiple physiological and pharmacological properties (pain relief, cancer prevention, and beneficial cardiovascular, and gastrointestinal effects), capsaicin has recently engrossed considerable attention for its antimicrobial and anti-virulence activity (Srinivasan, 2016 ▶). In this study, we examined different concentrations of capsaicin as an antivirulence agent to inhibit *V. cholerae* toxin production. We further examined whether the effect is dose-dependent or not.

## Materials and Methods

**Ethics statement**

This study was approved by the Ethics Committee of Shahid Beheshti University of Medical Sciences “IR.SBMU.RAM.REC.1394.443”.

**Bacterial strains**

Two standard strains namely, *V. cholerae *serogroup O1 serotype Ogawa ATCC 14035 and *V. cholerae* O1 serotype serotype Inaba PTCC 1611 were used in this study. The standard strains of *V. cholerae* ATCC 14035 was a kind Gift from Dr. Ali Hashemi (Shahid Beheshti University of Medical Sciences, School of Medicine, Department of Medical Microbiology) and *V. cholerae* PTCC 1611 was purchased from Iranian Research Organization for Science and Technology (IROST). 

**Minimum Inhibitory Concentration (MIC)**

To determine the lowest concentration of capsaicin (CAS Number 404-86-4; Sigma-Aldrich) that can kill the tested standard strains, Minimum Inhibitory Concentration (MIC) was determined by broth microdilution method according to the guidelines of Clinical Laboratory Standards Institutes (CLSI, 2012 ▶). Briefly, microbial inoculums in Mueller–Hinton broth (Merck*,* Darmstadt, Germany) were adjusted to a final concentration of 0.5 on the McFarland scale and diluted (1:20). Ten microliter of each inoculum was added to wells containing 100 µl of Muller-Hinton broth and capsaicin (with defined concentrations). After 24 hours of incubation at 37 ^o^C, microbial growth for each treatment was evaluated. 

**DNA extraction, PCR and sequencing**

Total DNA was extracted by the phenol-chloroform method as previously described (Shakibaie et al., 2008 ▶). PCR assay was accomplished to confirm the presence of *ctx*A, *ctx*B, *zot* and *rec*A genes. Specific PCR primers used in this experiment are shown in Table1. Thermal profiles were as follows: denaturation, annealing, and elongation temperatures and time periods were 94°C for 45s, 58°C for 45s, and 74°C for 45s, respectively, for 36 cycles, using a thermal cycler (Mastercycler gradient, Eppendorf). PCR products were analyzed by gel electrophoresis using 1% agarose gel; after staining with ethidium bromide, visible bands of proper size on the agarose gel, were read as positive results using a gel documentation system. To confirm the DNA sequence of each amplified gene, PCR products were sequenced (Bioneer Company, Korea). The nucleotide sequences were analyzed by Finch TV software and compared with sequences in the GenBank using the NCBI basic local alignment search tool (www.ncbi.nlm.nih.gov/BLAST).

**RNA extraction, cDNA synthesis and RT-PCR**

The two standard strains were grown overnight in modified yeast extract peptone (MYEP) medium at 37 °C. Based on MIC results, the bacteria were cultured in the presence of concentrations that did not affect bacterial growth. Bacteria were treated with 200, 100, 50, 10 and 1 µg/ml of capsaicin in DMSO that were prepared in MYEP medium. One extra tube which lacked capsaicin, was used as a control. Cultures were then incubated under a shaking condition for 24 hr. Following overnight incubation, Easy-BLUE™ Total RNA Extraction Kit (Cat. No. 17061) was used to extract total RNA from each inoculum. To avoid DNA contamination, the isolated RNA was further treated with DNase I, RNase-free (Pub. No. MAN0012000) according to the manufacturer’s guidelines. The integrity and purity of the extracted RNA were verified by NanoDrop spectrophotometer (Biochrom WPA Biowave II, UK) and on 0.8% agarose gel, prior to cDNA synthesis. The reverse transcription was carried out using the Maxime™ RT PreMix (iNtRON Biotechnology, Inc*.*) according to the manufacturer’s instruction. Briefly, cDNA was synthesized using 2µl of RNA at 45°C for 60 min, followed by incubation at 95 °C for 5 min using a thermal cycler (Master cycler gradient, Eppendorf). Capsaicin was dissolved in 2% DMSO during use.

**Real-Time PCR**

Real-time PCR was carried out using the synthesized cDNA and AccuPower® 2X GreenStar Master Mix Solution (Bioneer Inc.) by Rotor Gene 6000 RT*-*PCR Machine. Each set of primers are shown in Table 1. The housekeeping gene *rec*A expression level was used as an internal control and compared with that of the non-treated bacterial cultures. The relative expression in comparison with the internal control was analyzed according to (Chatterjee et al., 2010 ▶). Gene expression was normalized against *rec*A (housekeeping gene) and assigned as ΔCt values. Comparison of gene expression between control and treated samples was made by subtraction of control ΔCt values from treatment ΔCt values to give a ΔΔCT value. The relative gene expression was calculated using 2^- ΔΔ^^CT^ formula and normalized against controls. 

**Statistical analysis**

Student’s two-sample t-test was used in EXCEL to analyze statistical differences between the groups. A p-value of <0.05 was considered significant.

## Results

**Figure 1:**
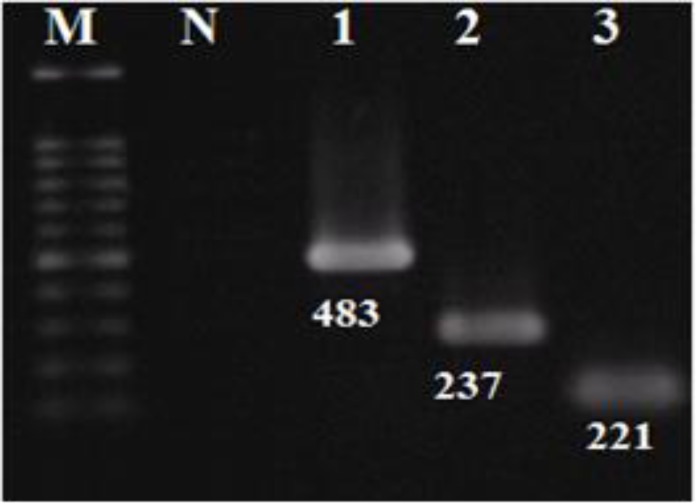
PCR amplification of *ctx*A, *ctx*B and *zot* genes of *V. cholerae*. Lane M: DNA size marker. Lane 1: zot gene, Lane 2: *ctx*B, and Lane 3: *ctx*A

Initially, two standard strains *V. cholerae *serogroup O1 serotype Ogawa ATCC 14035 and *V. cholerae* O1 serotype Inaba PTCC 1611 were selected to determine the effect of capsaicin on *V. cholerae* CT and ZOT production. Results of MIC test showed that 200 mg/mL of capsaicin in 2% DMSO could inhibit the growth of the two standard type strains of *V. cholerae* without killing them (2% DMSO alone did not show inhibitory effect on bacterial growth). Strains were positive for *ctx*A, *ctx*B, *zot* and *rec*A genes as assessed by PCR and electrophoresis (Figure 1). The results were confirmed by sequencing PCR products. The nucleotide sequence data reported in this paper have been submitted to the GenBank sequence database and assigned accession numbers KF551993 for *ctx*A, KU646842 for *ctx*B and KM094183 for *zot *gene.

**Table 1:** Primers used for PCR and Real-Time PCR

**Gene**	**PCR primers**	**Product size**	**Real-Time PCR primers**	**Product size**
***ctx*** **A**	F 5’-AGATTCTAGACCTCCTGATG-3’ R 5’-GAGTACCTCGGTCAAAGTAC-3’	221bp	F 5’-AGATTCTAGACCTCCTGATG-3’ R 5’-GAGTACCTCGGTCAAAGTAC-3’	83
***ctx*** **B**	F 5’-CACATGGAACACCTCAAAAT-3’ R 5’-GATATGCAATCCTCAGGGTA-3’	237	F 5’-TGCACATGGAACACCTCAAA-3’ R 5’-AGCCATCTCTCTTTTTCCAGC-3’	124
***zot***	F 5’-CTGCCTAACCACGCCTAACA-3’ R 5’-TGGACACAAAGCCGACCAAT-3’	483	F 5’- GAGCTTTGAGGTGGCTTTTG-3’ R 5’- GGTAAACTTTGCCCCTAGCC-3’	160
***rec*** **A**	F 5’-CAATTTGGTAAAGGCTCCATCAT-3’ R 5’-CCGGTCGAAATGGTTTCTACA-3’	71	F 5’-CAATTTGGTAAAGGCTCCATCAT-3’ R 5’-CCGGTCGAAATGGTTTCTACA-3’	71

**Figure 2:**
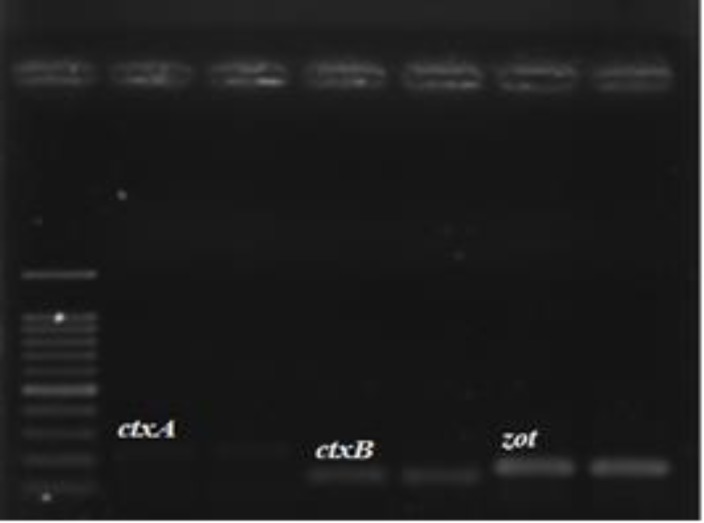
RT–PCR products electrophoresis on 1% agarose gel

In order to reach the appropriate annealing temperature, RT–PCR was performed. The result of RT-PCR products electrophoresis on 1% agarose gel is shown in (Figure 2).

The expression level of *ctx*A, *ctx*B and *zot* genes in the tested strains was reduced by capsaicin in a dose-dependent manner (p≤0.05) (Tables 2-4).

**Table 2:** Effect of capsaicin on decrease of expression level of *ctxA* gene in *Vibrio cholera*

**Standard strain**	**Concentrations of ** **Capsaicin**				
	**1** **(μg/ml)**	**10 (μg/ml)**	**50** **(μg/ml)**	**100 (μg/ml)**	**200 (μ** **g/ml)**
***Vibrio*** ***cholerae*** **ATCC14035**	1.11	1.23	1.60	7.10	7.16
***Vibrio*** ***cholerae* PTCC1611**	0.34	1.20	1.99	2.70	2.78

**Table 3:** Effect of capsaicin on decrease of expression level of *ctxB* gene in *Vibrio cholera*

**Standard strain**	**Concentrations of ** **Capsaicin**				
	**1** **(μg/ml)**	**10 (μg/ml)**	**50 (μg/ml)**	**100 (μg/ml)**	**200 (μ** **g/ml)**
***Vibrio*** ***cholerae*** **ATCC14035**	1.02	0.88	1.70	5.42	6.22
***Vibrio*** ***cholerae* PTCC1611**	0.87	1.29	2.07	4.40	5.90

**Table 4:** Effect of capsaicin on decrease of expression level of *zot* gene in *Vibrio cholerae*

**Standard strain**	**Concentrations of ** **Capsaicin**				
	**1** **(μg/ml)**	**10 (μg/ml)**	**50** **(μg/ml)**	**100 (μg/ml)**	**200 (μ** **g/ml)**
***Vibrio*** ***cholerae*** **ATCC14035**	1.11	1.14	1.51	1.62	2.2
***Vibrio*** ***cholerae* PTCC1611**	0.03	0.03	0.05	1.45	1.75

## Discussion

Capsaicin is the pungent fraction of red chili that showed great inhibitory effect against cholera toxin and Zot in the tested standard type strains of *V. cholerae*. The development of antivirulence agents, which are compounds that disarm bacteria rather than killing them, gave new hope in the struggle against antibiotic resistant bacteria alone or in combination with antibiotics. Due to the alarming rising trend of antibiotic resistance, there is a global call for finding new therapeutic approaches. A number of synthetic compounds have been tested for their possible potential to inhibit virulence factors in different strains of bacteria. toxtazin A and toxtazin B and B´ for example were found to act as inhibitors of CT transcription and toxin-coregulated pilus (TCP) (Anthouard and DiRita 2013 ▶). Compound 4 - a 4-alkylquinolin-2 (1*H*)-one scaffold- was synthesized and tested to inhibit pyocyanin production by *Pseudomonas aeruginosa.* It was shown that this compound reduced the level of pyocyanin production by *P. aeruginosa* by inhibiting quorum sensing between the bacteria (Morkunas et al., 2016 ▶). Natural compounds have always been a product of interest because of their safeness, fewer side effects, enormous variety and plentiful bioactive constituents which make them an appropriate candidate in search for effective therapeutic options (Lavecchia et al., 2013 ▶). Some natural compounds have already been tested against *V. cholerae*. Red chilli methanol extract and capsaicin at their sub-MIC concentration drastically inhibited CT production by *V. cholerae* strains (Chatterjee et al., 2010 ▶). Additionally, red bayberry (*Myrica rubra*) extract was shown to have inhibitory effects on growth and virulence gene expression of *V. cholerae *(Zhong, Yu and Zhu 2008 ▶). Also, Kalia and et al. showed that capsaicin significantly reduced the MIC of ciprofloxacin for *S. aureus* strains (Kalia et al. 2012 ▶). A recent study showed that anethole -a component of sweet fennel seed- could inhibit cholera toxin production by *V. cholerae* at its sub-MIC concentrations (Zahid et al., 2015 ▶). Since any kind of antimicrobial agent that target the viability of bacteria is likely to impose a selective pressure on development of antimicrobial resistance and also affects host natural flora, there is a growing trend to search for compounds that inhibit bacterial virulence factors rather than killing them (Clatworthy, Pierson and Hung, 2007 ▶). In our study, capsaicin at its sub-MIC concentrations, could reduce the expression levels of toxin genes in the tested *V. cholerae* standard strains (Table 1-3). Herein, we demonstrated that capsaicin exhibits great antivirulence activities by decreasing the expression of *V. cholerae* toxin-encoding genes. In case of resistant *V. cholerae* we would be able to propose this compound to control the bacterium. Our findings showed a dose-response function between the concentration of capsaicin and the expression level of *ctx*A, *ctx*B and *zot* genes.

Ginger extract as a herbal supplement was shown by other studies to prevent cholera-like diarrhea caused by* Escherichia coli* in developing countries (Nataro and Kaper, 1998). According to the WHO reports , in the past few years there is an increasing trend in cholera outbreaks (Muanprasat and Chatsudthipong, 2013 ▶). In addition, the size of cholera outbreaks is expected to gradually increase due to the global warming and lower immunity of humans( (Trærup, Ortiz and Markandya, 2011 ▶). With persistent global warming, cholera is speculated to continue to rise in the future (McMichael, Barnett and McMichael, 2012 ▶). Moreover, losing our antibiotic choices due to the growing trend of antibiotic resistance has exacerbated the situation and highlights the need to investigate alternative drugs like antivirulence agents. Additional studies are required to determine the efficacy of capsaicin in treating antibiotic-resistant bacteria. Investigating other derivatives of capsaicin may introduce new compounds with fewer side effects and improved therapeutic profile to be used as an antivirulence agent alone or as an adjuvant in combination with antibiotics for treatment of drug-resistant bacteria (Marini et al., 2015 ▶). 

Together, these results highlight the need to search for compounds that inhibit the virulence factors rather than killing the whole bacterium. Based on the results of this study, we found that capsaicin might suppress the expression of *ctx*A, *ctx*B (encoding cholera toxin) and also *zot* (which encodes zonula occludens toxin) genes in *V. cholerae*. These findings may lead to development of new antibacterial compounds based on natural products, against multidrug resistant bacterial pathogens including *V. cholerae*. There are several unknown aspects of antivirulence agents; also, numerous compounds are yet to be studied for their possible effects on *V. cholerae* virulence properties.
